# A comprehensive nano-interpenetrating semiconducting photoresist toward all-photolithography organic electronics

**DOI:** 10.1126/sciadv.abg0659

**Published:** 2021-06-18

**Authors:** Renzhong Chen, Xuejun Wang, Xin Li, Hongxiang Wang, Mingqian He, Longfei Yang, Qianying Guo, Shen Zhang, Yan Zhao, Yang Li, Yunqi Liu, Dacheng Wei

**Affiliations:** 1 State Key Laboratory of Molecular Engineering of Polymers, Department of Macromolecular Science, Fudan University, Shanghai 200433, China.; 2 Institute of Molecular Materials and Devices, Fudan University, Shanghai 200433, China.; 3 Corning Incorporated, Corning, NY 14831, USA.; 4 Institute of Chemistry, Chinese Academy of Sciences, Beijing 100190, China.

## Abstract

Owing to high resolution, reliability, and industrial compatibility, all-photolithography is a promising strategy for industrial manufacture of organic electronics. However, it receives limited success due to the absence of a semiconducting photoresist with high patterning resolution, mobility, and performance stability against photolithography solution processes. Here, we develop a comprehensive semiconducting photoresist with nano-interpenetrating structure. After photolithography, nanostructured cross-linking networks interpenetrate with continuous phases of semiconducting polymers, enabling submicrometer patterning accuracy and compact molecular stacking with high thermodynamic stability. The mobility reaches the highest values of photocrosslinkable organic semiconductors and maintains almost 100% after soaking in developer and stripper for 1000 min. Owing to the comprehensive performance, all-photolithography is achieved, which fabricates organic inverters and high-density transistor arrays with densities up to 1.1 × 10^5^ units cm^−2^ and 1 to 4 orders larger than conventional printing processes, opening up a new approach toward manufacturing highly integrated organic circuits and systems.

## Teaser

A semiconducting photoresist contributes to precise and reliable manufacture of highly integrated organic electronics.

## INTRODUCTION

Photolithography, as a high-precision manufacturing technology, has been widely applied in the semiconductor industry, rendering it the capability to refine the features following the Moore’s law for half a century (*1*, *2*). Until now, silicon-based electronics comes to 5-nm nodes. As a comparison, the miniaturization of organic electronics is far behind silicon electronics because of fundamental limitations (e.g., resolution and reliability) of solution-based manufacture processes (*3*, *4*). The highest transistor density of organic circuits fabricated by printing technologies is 60 units cm^−2^, equivalent to 19.7 pixels per inch (PPI) (*5*–*7*). To address this issue, all-photolithography process holds great promise for further miniaturization of organic electronics. It patterns organic semiconductors (OSCs), dielectrics, and conductors with higher resolution, reliability, as well as compatibility to current microelectronic manufacturing industry (*8*–*10*). However, in the present, there still lacks an OSC that is deeply compatible with the all-photolithography process (*11*, *12*).

All-photolithography requires a “semiconducting photoresist” that has high patterning resolution, mobility, as well as performance stability against solution processes. The semiconducting photoresist not only simplifies the photolithography procedure (see fig. S1) but also circumvents the compatibility issue between OSC and photolithography. Various photocrosslinkable OSCs have been developed, which are designed mainly on the basis of a side-chain cross-linking strategy (*13*, *14*). Unfortunately, the photocrosslinkable OSCs do not equal semiconducting photoresists, because they fail to meet these requirements. First, the cross-linked side chains intrapenetrate with the conjugated backbones and disturb π-π stacking of the conducting phase, inevitably sacrificing electrical performance with mobilities rarely above 0.5 cm^2^ V^−1^ s^−1^ (*15*–*31*). Second, the side-chain cross-linking of conjugated polymers leads to challenges in achieving submicrometer patterning resolution comparable to that of photoresists made of small molecules or oligomers, such as SU-8 (*32*). The intrapenetrating structure results in thermodynamic metastable π-π stacking, which makes the electrical properties susceptible to solvent. Subsequent solution processes of photolithography (e.g., developing and stripping) markedly degrade the device performance and homogeneity, which cannot be tolerated in practical applications of integrated organic circuits. Therefore, existing photocrosslinkable OSCs cannot meet the requirements of all-photolithography, hampering the continual miniaturization of organic electronics along with the rapid development of the microelectronic manufacturing industry.

Here, we develop a comprehensive semiconducting photoresist (SP-1) with compatibility to all-photolithography. It has a nano-interpenetrating structure, which enables submicrometer patterning resolution and compact π-π stacking, overcoming the limitations of photocrosslinkable OSCs. Organic thin-film transistors (OTFTs) fabricated by photolithography exhibit not only enhanced mobilities up to 1.64 cm^2^ V^−1^ s^−1^ but also excellent stability of electrical performance against solution processes in photolithography. On the basis of SP-1, we realize all-photolithography and fabricate organic logic circuit elements, OTFT arrays, and flexible OTFTs precisely, efficiently, and reliably with a density up to 1.1 × 10^5^ units cm^−2^ (equivalent to 848 PPI).

## RESULTS

### Semiconducting photoresist

**Fig. 1:**
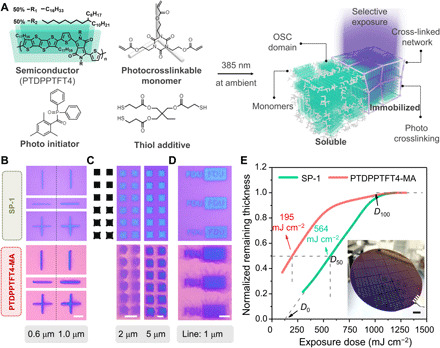
Photolithography of SP-1. (**A**) Schematic diagram showing the photocrosslinking of SP-1. (**B** to **D**) Optical microscope (OM) images of fine patterns with different designed sizes (exposure doses <600 mJ cm^−2^). (**E**) Contrast curves of SP-1 and PTDPPTFT4-MA. The inset is a photograph of patterned SP-1 transistor arrays on a 4-inch wafer. Photo credit: Renzhong Chen, Fudan University. Scale bars, 5 μm in (B to D) and 1 cm in (E).

As shown in Fig. 1A, SP-1 comprises a donor-acceptor structure-based p-type semiconductor polymer [poly(tetrathienoacene-diketopyrrolopyrrole) (PTDPPTFT4)], a cross-linkable monomer (tris[2-(acryloyloxy)ethyl] isocyanurate), a radical photoinitiator [2,4,6-trimethylbenzoyldiphenylphosphine oxide (TPO)], and a thiol additive [trimethylolpropane tris(3-mercaptopropionate)]. PTDPPTFT4 contains a highly planar conjugated backbone, and 50% diketopyrrolopyrrole (DPP) is grafted with a branched alkyl side chain. Thus, it provides highly conducting channel with mobility around 1 cm^2^ V^−1^ s^−1^ and moderate solubility in the photoresist (*33*,*34*). The cross-linkable monomer with three linkage sites has photocrosslinking efficiency as well as compatibility to PTDPPTFT4, which avoids macroscale phase separation and allows formation of the nano-interpenetrating structures (*35*). Figures S2 and S3 reveal the effects of photocrosslinkable component type and content in electrical performance and aggregation structure, and SP-1 has the optimized OSC/monomer weight ratio of 2:1. The photoinitiator is sensitive to a 385-nm ultraviolet (UV) light of our mask aligner, which can initiate the radical polymerization reaction of acrylate C═C. The thiol additive plays the role of radical transferring agent and stabilizer to make sure efficient photocrosslinking reaction against oxygen at ambient conditions (*36*). As a comparison, we synthesize three photocrosslinkable PTDPPTFT4 polymers containing 15, 25, and 50% methacrylate (MA)–functionalized side chains, and the one containing 25% MA is termed PTDPPTFT4-MA (fig. S4). To promote the photocrosslinking of PTDPPTFT4-MA, we also use the photoinitiator and the thiol additive.

### Photolithographic performance

Geometric structures, such as lines, squares, and texts, are patterned with different exposure doses (see Fig. 1, B to D, and fig. S5A). The minimum width of the lines (fig. S6) is designed to be 0.6 μm, close to the resolution limit of the 385-nm light source. We design the square patterns with the bulgy corners, and the SP-1 inherits the distinctive pattern details very well with uniform thickness and steep sidewalls (see fig. S5, B and C). The well-defined positive and negative asperities of 1-μm line-width “FDU” texts show the potential of SP-1 in manufacturing complex fine patterns with micro- or submicrometer resolution. In contrast, PTDPPTFT4-MA is not capable of yielding these high-resolution patterns with a large number of residues. We evaluate the patterning reliability of SP-1 in photolithography. Hundreds of OTFT devices are fabricated on a 4-inch wafer (Fig. 1E), and no residue exists.

The sensitivity (S) and contrast (γ) are two quantitative parameters of photoresist, which can be extracted by plotting the film thickness as a function of exposure dose (*37*). γ is calculated by γ =$\;{\left[\text{log}(\frac{D_{100}}{D_{0}})\right]}^{-1}$, where *D*_100_ and *D*_0_ correspond to the exposure doses for complete reaction and initial reaction, respectively. Typically, to fabricate submicrometer patterns, one of the prerequisites is high γ greater than 1. According to the contrast curves (Fig. 1E), S (*D*_50_) and γ of SP-1 are 564 mJ cm^−2^ and 1.09, respectively. PTDPPTFT4-MA presents a rapid rise trend of remaining thickness when the doses increased from 0 to 100 mJ cm^−2^, γ is below 0.9, and S is only 195 mJ cm^−2^. The high sensitivity and low resolution of PTDPPTFT4-MA is attributed to the side-chain cross-linking of semiconducting polymers, which remarkably decreases solubility even after slight exposure. In the case of SP-1, the monomers and oligomers (doses <121 mJ cm^−2^) are soluble; thus, efficient dissolution of unexposed photoresists occurs, forming sharp edges with high resolution.

### Mobility and performance stability

**Fig. 2:**
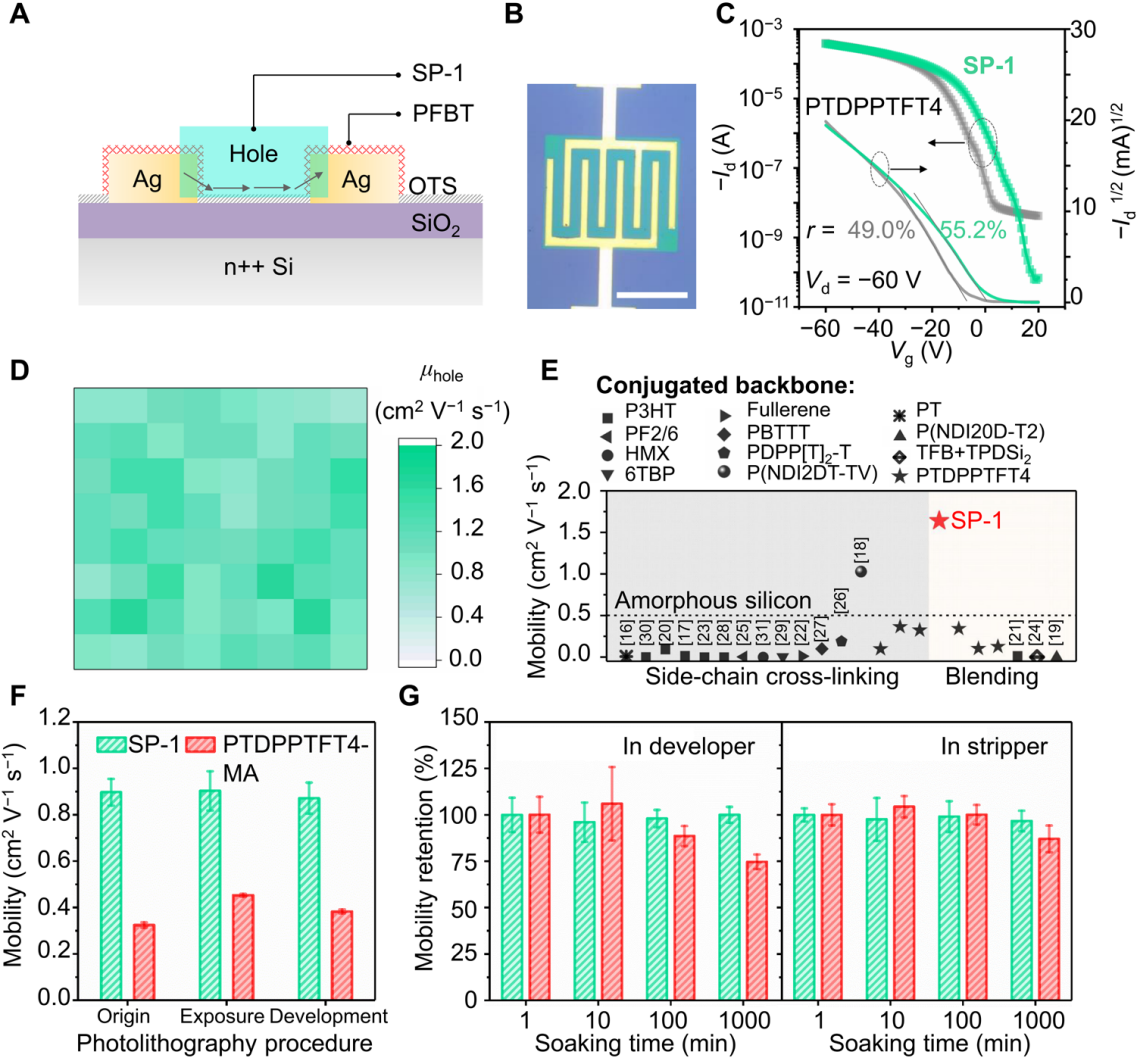
Electrical performance and stability. (**A**) Schematic configuration, (**B**) OM image, (**C**) transfer curves, and reliability factors (*r*) of OTFT devices. (**D**) Two-dimensional (2D) mapping for mobilities of 8 × 8 OTFT arrays based on patterned SP-1. (**E**) Comparison of the highest mobilities of patterned SP-1 and photocrosslinkable OSCs reported previously. (**F**) Mobilities of SP-1 and PTDPPTFT4-MA during photolithography procedures. (**G**) Mobility retentions after soaking in developer and stripper for different times. Scale bar, 500 μm.

The electrical performance of SP-1 is examined using bottom-gate bottom-contact (BGBC) configured OTFT devices (Fig. 2A). To improve the device performance, we modify the SiO_2_/Si wafer and Ag electrodes with self-assembled monolayers of octadecyltrichlorosilane (OTS) and pentafluorobenzenethiol (PFBT), respectively (*38*). The representative optical microscope (OM) image (Fig. 2B) shows that the patterned SP-1 exactly resides on design region. The transfer and output curves (Fig. 2C and fig. S7) present similar semiconducting properties of SP-1 to that of pristine PTDPPTFT4 film with a slightly enhanced mobility around 1 cm^2^ V^−1^ s^−1^ (fig. S8) and an 11-fold higher on/off ratio (>10^6^). Because of the contact resistance between semiconductor and bottom electrodes, nonlinearity of transfer characteristics exists (Fig. 2C). The reliability factors (*r*) of slopes are calculated to be 49.0 and 55.2% for pristine PTDPPTFT4 and SP-1, respectively (*39*). As a comparison, cross-linkable side chains reduce the electrical performance. The mobilities of the photocrosslinkable PTDPPTFT4 with 15, 25, and 50% MA are only 33.0, 37.2, and 9.4% of that of pristine PTDPPTFT4 film, respectively. We fabricate 8 × 8 OTFT array on a 4 cm by 4 cm wafer by photolithography. Overall, 100% devices (see Fig. 2D) work well with high uniformity of electrical performance. Transfer curves of 64 devices overlap well, as shown in fig. S9. The hole carrier mobilities of 64 transistors have a narrow distribution, with an average of 1.11 cm^2^ V^−1^ s^−1^ and a small coefficient of variation down to 20.8%. The mobility reaches up to 1.64 cm^2^ V^−1^ s^−1^, which is the highest value of photocrosslinkable OSCs reported until now (Fig. 2E) (*16*–*31*).

Besides the mobility, the stability of electrical performance against photocrosslinking and any solution processes (e.g., developing and stripping) in photolithography is of great importance for its practical applications. Our measurements (Fig. 2F and fig. S10) show that the mobilities (> 0.9 cm^2^ V^−1^ s^−1^) of SP-1 are highly stable with variations less than 3.5% after 400 mJ cm^−2^ UV exposure and soaking in developer [chlorobenzene and 1,4-dioxane (v/v 9:1) mix solvent] for 1 min, respectively. *V*_th_ (fig. S10) of SP-1 slightly increases by ~1.5 V after exposure because of the compositional changes induced by the cross-linking reaction. After development, *V*_th_ of SP-1 remains unchanged. Long-term duration against developer and stripper (Remover PG) were performed to examine the stability against multiple photolithography processes. The developed SP-1 maintains almost 100% mobility even after 1000-min soaking in developer and stripper (Fig. 2G). It renders SP-1 the capability of fabricating the BGTC structured OTFT (see fig. S11) with an uncompromised mobility by a two-step photolithography. Without nano-interpenetrating structure, the mobilities of PTDPPTFT4-MA vary from 0.325 to 0.453 cm^2^ V^−1^ s^−1^ after UV exposure and decrease to 0.383 cm^2^ V^−1^ s^−1^ after developing, with variations more than 17.8%. The mobility of side-chain cross-linked PTDPPTFT4-MA is larger than that of uncross-linked PTDPPTFT4-MA, owing to the decreased amount of active groups (*21*) and the tightened molecular packing (*40*); however, the mobility is still smaller than that of cross-linked SP-1. In photolithography procedures, PTDPPTFT4-MA has larger variations of *V*_th_ and subthreshold slope than SP-1 (see fig. S10). In addition, the mobility of PTDPPTFT4-MA decreases to 74.6 and 87.1% after soaking for 1000 min in developer and stripper, respectively, consistent with previous literatures *(**12**)*.

### Aggregation structure and mechanism

**Fig. 3:**
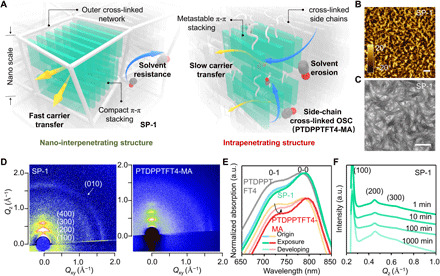
Aggregation structures in photolithography. (**A**) Schematic illustrations of nano-interpenetrating and intrapenetrating structures. (**B** and **C**) AFM and TEM images of developed SP-1. (**D**) 2D GIXRD patterns of photocrosslinked SP-1 and PTDPPTFT4-MA. (**E**) UV-vis spectra of PTDPPTFT4 as well as SP-1 and PTDPPTFT4-MA during photolithography procedures. (**F**) 1D GIXRD curves of developed SP-1 after soaking in developer for different times. Scale bars, 500 nm. a.u., arbitrary units.

The nano-interpenetrating structure (Fig. 3A) and its nanoconfinement effect enable higher mobilities and better performance stability compared with the intrapenetrating structure. The aggregated structures of SP-1 are characterized by atomic force microscopy (AFM) (Fig. 3B and fig. S12) and transmission electron microscopy (TEM) (Fig. 3C and fig. S12). The results (fig. S12) show that the pure PTDPPTFT4 and the developed PTDPPTFT4-MA form a homogeneous film without bundle-like structures, while SP-1 has a different aggregation structure. AFM image in phase mode reveals that the developed SP-1 has a phase separation in nanoscale. TEM image shows that the material has a uniformly interpenetrating structure with bundle-like aggregates (*41*), obtaining a continuous PTDPPTFT4 phase.

Two-dimensional (2D) grazing incidence x-ray diffraction (GIXRD) patterns show the pristine PTDPPTFT4 (fig. S13A) and PTDPPTFT4-MA (Fig. 3D) have an edge-on stacking configuration with well-defined (*X*00) and (010) diffraction along the out-of-plane and in-plane directions, respectively. The (010) diffraction of SP-1 features a ring-like profile with enhanced face-on alignment, which indicates a uniform distribution of π-π stacking in 3D directions, forming a 3D charge-transfer network (*42*). GIXRD results (fig. S13 and table S1) show that the lamellar spacing (27.6 Å) and π-π stacking distances (3.678 Å, in-plane direction) of cross-linked SP-1 are smaller than those of pristine PTDPPTFT4 and PTDPPTFT4-MA. Meanwhile, the coherence length of cross-linked SP-1 is estimated to be 6.8 nm for (010) diffraction, larger than those of pristine PTDPPTFT4 (4.9 nm) and cross-linked PTDPPTFT4-MA (6.2 nm). These results indicate that the cross-linked SP-1 has a more compact and higher-ordered π-π stacking.

Normalized UV–visible (vis) spectra (Fig. 3E) reveal that the developed SP-1 has an increased *A*_0-0_/*A*_0-1_ value with a 4.5-nm red shift, where *A*_0-0_ and *A*_0-1_ are the absorption intensity of 0-0 peak and 0-1 peak, respectively, indicating that the semiconducting phase has larger conjugated length and higher-ordered arrangement compared with the pristine PTDPPTFT4 (*43*), consistent with the GIXRD results. This phenomenon is attributed to the nanoconfinement effect of photocrosslinking networks (*41*, *44*), as higher monomer content gives rise to a larger *A*_0-0_/*A*_0-1_ value (see fig. S3A). Therefore, the continuous 3D charge-transfer network (figs. S2 and S3, E and F), compact molecular stacking, and large conjugated length lead to high mobility of the developed SP-1.

We characterize the aggregation structures of SP-1 throughout the photolithography processes. The nanosized phase separation occurs with bundle-like structures before photocrosslinking (fig. S14). After photolithography and 1000-min soaking in developer, not only the morphology (fig. S14) but also the 0-1 peaks in UV-vis spectrum (Fig. 3E and fig. S15) and the (*X*00) peaks (Fig. 3F) in 1D GIXRD maintain without remarkable changes. The robust aggregation of SP-1 is attributed to the nano-interpenetrating structure. First, the microphase separation allows SP-1 to form continuous semiconducting phases. Second, photocrosslinked networks surround the micro domains of the semiconducting polymers that lock and protect the semiconducting phases. The nanoconfinement effect of the networks tightens the π-π stacking with high thermodynamic stability, thus rendering semiconducting phases the capability of resisting critical photolithography conditions, such as UV exposure (fig. S16) and solution erosion (*44*, *45*). In the case of PTDPPTFT4-MA, cross-linked side chains intrapenetrate in the aggregates, which disturb π-π stacking and cause a thermodynamically metastable molecular arrangement. As approved by the UV-vis absorption spectra (Fig. 3E and fig. S15), 0-1 peak of PTDPPTFT4-MA varies appreciably throughout photolithography and long-time soaking. In this manner, notable variation and degradation of the mobility occur.

The thermodynamically stable structure of SP-1 is evaluated by measuring the mobility at temperatures (fig. S17) (*41*, *46*). The cross-linked SP-1 exhibits high stability comparable with the pristine PTDPPTFFT4, and the mobility remains stable even at 230°C. As a comparison, the mobility of cross-linked PTDPPTFT4-MA decreases when the temperature is above 200°C. Therefore, the nano-interpenetrating structure achieves high mobility and excellent performance stability, overcoming the bottleneck of OSCs in photolithography.

### All-photolithography of organic devices and circuits

**Fig. 4:**
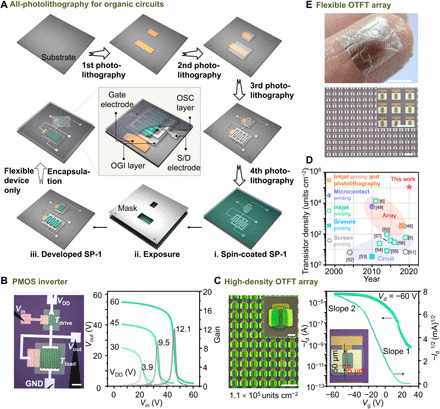
All-photolithography based on SP-1. (**A**) Schematic of all-photolithography process for organic circuits. (**B**) OM image, voltage transfer curves, and signal inverter gain of as-fabricated PMOS inverter. (**C**) OM images of as-fabricated OTFT array with a density of 111343 units cm^−2^ and a transfer curve of the device. The mobilities are 1.276 cm^2^ V^−1^ s^−1^ (for slope 1) and 0.039 cm^2^ V^−1^ s^−1^ (for slope 2) with reliability factors of 27.1 and 891%, respectively. (**D**) Comparison of the transistor density of organic integrated circuits and arrays. (**E**) OM images and photograph of an OTFT array on flexible substrate before and after releasing. Photo credit: Renzhong Chen, Fudan University. Scale bars, 500 μm in (B, bottom of E), 50 μm in (C), 10 μm in (the inset of C), 5 mm (top of E). GND, ground.

Because of high patterning resolution, mobility, and stability, SP-1 realizes all-photolithography by a simple and reliable manner. The all-photolithography procedure with multistep exposures is displayed in Fig. 4A for fabricating a transistor with a buried gate electrode, which begins with a clean SiO_2_/Si substrate. Cr/Au electrodes were patterned as the bottom gates by first-step photolithography using a positive photoresist S1813. Then, a modified negative tone silicone photoresist SINR-3570PE-5.0 (termed photoresist 3570) was patterned by second-step photolithography as the organic dielectric (OGI) layer. The thickness and specific capacity of the dielectric layer measured by a scanning electron microscope (SEM) and LCR meter (figs. S18 and S19) are 342 nm and 4.96 nF cm^−2^. Subsequently, Cr/Ag electrodes were patterned as the source/drain (S/D) electrodes by third-step photolithography using the photoresist S1813. Last, SP-1 was used as the photoresist to fabricate the semiconducting channel by fourth-step photolithography.

A P-channel metal oxide semiconductor (PMOS) Zero-V_GS_-load logic (*47*) inverter (Fig. 4B and fig. S20) was fabricated with a drive transistor (*W*/*L* = 2000/50 μm) and a load transistor (*W*/*L* = 20,000/50 μm). The inverter gave the expected carrier transfer behavior with the same range of input and output voltages and outperformed a high gain of 12.1 at a supply voltage (*V*_DD_) of 60 V. The gain is comparable to that of the printed PMOS inverters (see table S2). To demonstrate the potential application in high-integrated organic devices, we fabricated transistor arrays (Fig. 4C, and figs. S21 and S22) with high densities of 4489, 10,314, 28,190, 52,068, and 111,343 units cm^−2^. The maximum is one to four orders higher than the transistor density (Fig. 4D) of organic integrated circuits and arrays fabricated by conventional printing processes (*5*, *6*, *48*–*57*). Owing to the 5 μm channel length, the transfer curve presents an obvious double-slope feature (*58*). The average hole mobilities of 20 transistors in the 4489 units cm^−2^ OTFT array are 0.696 cm^2^ V^−1^ s^−1^ (slope 1) and 0.067 cm^2^ V^−1^ s^−1^ (slope 2), and the highest value (in Fig. 4C) reaches 1.276 cm^2^ V^−1^ s^−1^ (27.1%, slope 1), indicating the uncompromised performance of SP-1 in all-photolithography. Moreover, flexible organic devices based on SP-1 can be manufactured by all-photolithography with cross-linked photoresist 3570 as both substrate and encapsulation layer. As shown in Fig. 4E and fig. S23, we constructed a flexible OTFT array, which can be attached conformably onto a finger. The flexible OTFT array with a high density of 4489 units cm^−2^ on a polyethylene naphthalate (PEN) film (fig. S24) has an average mobility of 0.471 cm^2^ V^−1^ s^−1^, and the device keeps 90.8% mobility after bending 1000 times, showing the potential in flexible organic electronics.

## DISCUSSION

This work demonstrates a nano-interpenetrating semiconducting photoresist. The submicrometer resolution approaches to the resolution limit of the mask aligner, allowing precise fabrication of highly integrated organic electronics. The density of OTFT arrays reaches 1.1 × 10^5^ units cm^−2^, one to four orders higher than that fabricated by conventional printing technologies (*5*–*7*), which meets the requirement of drive-transistor density in 848-PPI electronic papers. The average mobility is 1.11 cm^2^ V^−1^ s^−1^ with a maximum up to 1.64 cm^2^ V^−1^ s^−1^, comparable or even higher than that of amorphous Si, which renders SP-1 an attractive alternative of amorphous Si in flexible displays, organic logic circuits, and sensors for wearable applications. Moreover, the nano-interpenetrating structure gives rise to a stable semiconducting channel with improved molecular stacking and orientation, which enables SP-1 to maintain almost 100% mobility even soaking in developer or stripper for 1000 min, avoiding the inhomogeneity of device performance caused by photolithography solution processes. Thus, SP-1 solves the stability issue of side-chain photocrosslinkable OSCs and matches the requirement of all-photolithography. Besides semiconducting photoresists, the nano-interpenetrating structure can also be used to design functionalized photoresists customized for other applications, including but not limited to organic electronics, optoelectronics, and photonics.

There have been numbers of OSCs with mobilities higher than 1 cm^2^ V^−1^ s^−1^, which already satisfy the requirements of electrical performance in some applications. However, the major challenge of the application is how to precisely and reliably manufacture complicated OSC patterns. As a revolutionary manufacture technology, all-photolithography overcomes limitations of conventional solution-based manufacture and opens up new opportunities for continual miniaturization of organic electronics with high compatibility to current microelectronics manufacturing industry. Considering the rich accumulations of photolithography-based manufacturing technologies, all-photolithography, along with the compatible semiconducting photoresists, promises to narrow the technological gap between organic electronics and silicon-based electronics, paving a board avenue toward the industrialization of highly integrated organic electronics in the future.

## MATERIALS AND METHODS

### Composition of SP-1

SP-1 was composed of a polymer semiconductor (PTDPPTFT4, Corning Inc.), a cross-linkable monomer (tris[2-(acryloyloxy)ethyl] isocyanurate), a free radical photoinitiator [diphenyl[2,4,6-trimethylbenzoyldiphenylphosphine oxide (TPO)] and a thiol additive [trimethylolpropane tris(3-mercaptopropionate)], which were directly used without purification. PTDPPTFT4 and cross-linkable monomers (weight ratio 2:1) were dissolved in chlorobenzene (CB) to obtain a blending solution with a PTDPPTFT4 concentration of 20 mg ml^−1^. Before photolithography, photoinitiators [5 weight % (wt %) of monomers] and thiol additives (5 wt % of monomers) were added to the above blends. The control sample consisted of a PTDPPTFT4-backbone polymer semiconductor (PTDPPTFT4-MA; Corning Inc.), 3 wt % photoinitiator, and 1.5 wt % thiol additive, which dissolved in CB with a PTDPPTFT4-MA concentration (15 mg ml^−1^).

### Fabrication of OTFTs

An n-type heavily doped Si wafer with a 300-nm SiO_2_ layer (specific capacitance COX = 11 nF cm^−2^) served as a bottom-gate electrode and a dielectric layer, respectively. To fabricate BGBC structured OTFTs, 5/45-nm-thick Cr/Ag layers were thermally evaporated on the pristine SiO_2_/Si substrates through the photolithography process (using S1813 photoresist) as bottom S/D electrodes. After liftoff by stripper (Remover PG, MicroChem), the substrate with S/D electrodes was ultrasonic cleaned with heptane, ethanol, acetone, and chloroform. As-cleaned substrate was treated with OTS in a vacuum oven at a temperature of 120°C, forming an OTS self-assembled monolayer, and then sonicated in heptane, ethanol, and chloroform, successively to remove redundant OTS molecules. S/D electrodes were modified by soaking in ethanol solution of PFBT (v/v 1:1000) for 2 min and then rinsed in ethanol to remove extra PFBT. The substrate was dried at 130°C for 9 min. Ultimately, SP-1 solution (20 mg ml^−1^) was deposited on the as-treated substrate by spin coating.

Photolithography was achieved via the MicroWriter ML3 laser direct writing photoengraving machine (Durham Magneto Optics Ltd.). The simplified photolithography process (fig. S1) of SP-1 is described as follows: Spin-casted film was prebaked at 130°C for 2 min and then exposed by the mask aligner (385-nm UV light source) with a certain exposure dose (300 to 1200 mJ cm^−2^). Subsequently, the as-exposed film was soaked in a developer composed of CB and 1,4-dixane (v/v 9:1) for 1 min with violent shaking. Last, a post bake at 190°C for 10 min was carried out to complete photolithography. To fabricate BGTC-structured OTFT, the process was similar. The difference was that the semiconducting layer (SP-1) was fabricated before S/D electrodes.

### OTFT arrays and inverters

To fabricate buried gate electrodes, a SiO_2_/Si wafer coated with patterned photoresist S1813 (MICROPOSIT) was etched by 10% HF for 20 s before metal deposition (5-/25-nm Cr/Au). To match with the light source of the mask aligner, a modified photoresist SINR-3570PE-5.0 (Shin-Etsu, termed 3570) was used as dielectric, which was composed of SINR-3570PE-5.0 cyclopentanone solution (500 mg ml^−1^), 0.35 wt % photosensitizer (Irgacure ITX, BASF), and 0.35 wt % photoinitiator (Irgacure 290, BASF). The photoresist 3570 deposited upon gate electrode via spin coating with a rotation speed of 3000 rpm for 30 s and exposed by 385-nm UV light with a dose of 2400 mJ cm^−2^. After exposure, a postexposure bake was carried out at 170°C for 30 min to increase the degree of cross-linking. Then, the patterns can be obtained by developing at propylene glycol methyl ether acetate for 120 s. Subsequently, a 5-/60-nm-thick Cr/Ag S/D electrodes were patterned through the photolithography process and thermal evaporation. The semiconducting channel was patterned using SP-1 via a simplified photolithography process (fig. S1). Except for thermal evaporation, all processes, including photolithography, anneal, and spin coating, were performed under an ambient atmosphere.

### Flexible OTFT arrays

As the flexible substrate, photoresist SINR-3570PE-5.0 was first coated on a SiO_2_/Si wafer with a rotation speed of 3000 rpm for 30 s and then exposed by a 365-nm UV box for 3 min. After exposure, a postexposure bake was carried out at 170°C for 30 min. The gate electrodes were fabricated via photolithography (photoresist S1813) and metal deposition (5-/15-nm Cr/Au) without prior HF etching. The OGI layer, S/D electrodes, and channel layer were patterned layer by layer using a procedure similar to OTFT arrays and inverters. As an encapsulation layer, photoresist SINR-3570PE-5.0 was spin-coated top with a rotation speed of 3000 rpm for 30 s and exposed by the 365-nm UV box for 3 min. Subsequently, a postexposure bake was carried out at 130°C for 120 min. The flexible array was released from the SiO_2_/Si wafer via 40% HF etching and then washed by deionized (DI) water. Last, the flexible array floated on DI water can be transferred to PEN films and then dried at 115°C for 30 min.

### Characterization

Electrical characterization of the OTFTs was carried out by a Keithley 4200 semiconductor analyzer in ambient conditions. The capacitance of the dielectric film was measured using a TH2830 LCR meter. The samples were measured by an OM (Nikon), atomic force microscope (XE7, Park Systems), SEM (Thermo Fisher Scientific), transmission electron microscope (Tecnai G2 20 TWIN), and UV-vis absorption spectroscope (PerkinElmer Lambda750). The GIXRD data were obtained at beamline BL14B1 of the Shanghai Synchrotron Radiation Facility.

## SUPPLEMENTARY MATERIALS

Supplementary material for this article is available at http://advances.sciencemag.org/cgi/content/full/7/25/eabg0659/DC1
